# Budget impact analysis of insulin glargine vs. isophane protamine insulin treatment for patients with type-2 diabetes and severe hypoglycemia in Thailand

**DOI:** 10.3389/fpubh.2024.1415908

**Published:** 2025-01-14

**Authors:** Unchalee Permsuwan, Chaicharn Deerochanawong

**Affiliations:** ^1^Department of Pharmaceutical Care, Faculty of Pharmacy, Chiang Mai University, Chiang Mai, Thailand; ^2^Center for Medical and Health Technology Assessment (CM-HTA), Department of Pharmaceutical Care, Faculty of Pharmacy, Chiang Mai University, Chiang Mai, Thailand; ^3^Rajavithi Hospital, College of Medicine, Ministry of Public Health, Rangsit University, Bangkok, Thailand

**Keywords:** budget impact analysis, insulin glargine, NPH insulin, diabetes, Thailand

## Abstract

**Objective:**

This study aimed to assess the financial impact of different adoption rates of insulin glargine (IGlar) treatment compared to isophane protamine (neutral protamine hagedorn [NPH]) insulin treatment for patients with type-2 diabetes (T2D) and severe hypoglycemia in Thailand from the payer’s perspective.

**Methods:**

The budget impact analysis (BIA) model over a period of 5 years was used to estimate the net budget impact (NBI) of IGlar treatment by comparing the total budget expenditures under two scenarios: scenario 1 involved only NPH insulin and scenario 2 included the introduction of IGlar. The total budget included either the cost of insulin or a combination of the costs of insulin and the expense related to severe hypoglycemia. Scenario 2 started at 20% uptake of IGlar and a yearly increase of 20%. NBI was calculated as the difference between the total budgets of scenarios 1 and 2. NBI and one-way sensitivity analyses were conducted for evaluation.

**Results:**

Considering only the cost of insulin, the use of IGlar for patients with T2D and severe hypoglycemia resulted in a yearly average NBI of 174.9 million Thai baht (THB) (5.1 million USD). However, when the cost related to severe hypoglycemia was included, the total budget incurred from scenario 2 was less than that of scenario 1, leading to a negative NBI or cost savings.

**Conclusion:**

The NBI of IGlar adoption would be substantial when considering only the cost of insulin; however, the significant benefit of IGlar in terms of a lower rate of severe hypoglycemia compared with NPH insulin would clearly offset the additional cost of IGlar.

## 1 Introduction

Type-2 diabetes (T2D) is a rapidly rising public health issue globally. In 2021, T2D affected approximately 536.6 million people worldwide and is estimated to increase to approximately 783.2 million by 2045 (1). In Thailand, 6.1 million adults are affected by diabetes (1); according to the Fifth National Health Examination Survey, nearly 8.9 and 10.8% of Thai men and women, respectively, were affected by T2D, among which less than one-half (45.9 and 36.4%, respectively) received T2D treatment (2). Hypoglycemia is a risk for people with T2D being treated with insulin, with reported rates of severe hypoglycemia approximately 2.5 events per person per year (3). Severe hypoglycemic events may generate expensive hospitalization. Minor hypoglycemic events do not require hospitalization, but the occurrence of minor events in high frequency might result in substantial costs and lost productivity (4). Costs of severe hypoglycemia for outpatient visits and inpatient admissions in Thailand were approximately 3,102 THB (103 USD) and 74,532 THB (2,475 USD), respectively (5). Therefore, issues such as glycemic control, adverse events, convenience, and costs should be considered before selecting an appropriate insulin type for individuals with T2D.

In Thailand, insulin glargine (IGlar) is currently listed in the National List of Essential Medicine (NLEM) Category D, referring to medicines used only for particular indications and diseases. IGlar can be prescribed only for type-1 diabetes (T1D) under the conditions that patients have severe hypoglycemia or nocturnal hypoglycemia after using multiple daily human insulin injections (6). Human insulin is the primary type listed in the NLEM for T2D. The study of 36,793 patients with T2D from 1,018 healthcare facilities across Thailand reported that 22.80% were insulin users (7). Since IGlar is more expensive than human insulin, budget impact analysis (BIA) is required to provide economic evidence for the overall financial budget to decide on whether to extend IGlar use for T2D patients. Therefore, this study was conducted regarding the NLEM in Thailand to assess the financial impact of different adoption rates of IGlar treatment instead of conventional insulin treatment for patients with T2D and severe hypoglycemia.

## 2 Materials and methods

**Figure 1:**
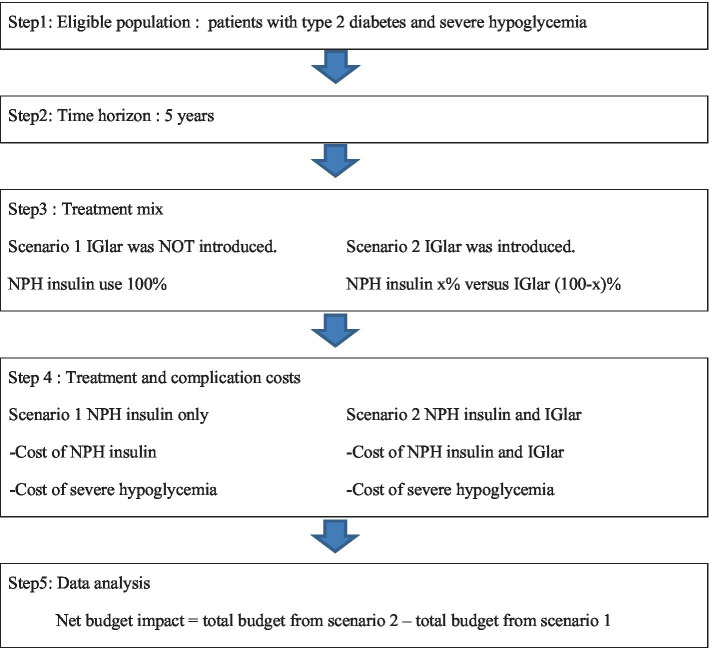
Framework of budget impact analysis. IGlar, insulin glargine; NPH, isophane protamine insulin.

The analytical framework for BIA analysis of IGlar uptake for patients with T2D and severe hypoglycemia is shown in Figure 1. In this study, conventional insulin is referred to as isophane protamine (NPH) insulin. BIA required epidemiologic data that included prevalence, incidence, mortality rate, and cost. All inputs were based on the published studies and the data from Thailand.

### 2.1 Study inputs

#### 2.1.1 Estimation of insulin glargine candidates

We began to estimate insulin users based on the market sale of NPH insulin in Thailand. The yearly average NPH insulin use from 2017 to 2021 was 844,472,700 units. Based on the expert’s opinion, daily insulin dosage was 25 units/patient, resulting in insulin use of 9,125 units/patient/year. Therefore, currently, the total number of NPH insulin users sums up to 92,545 patients (844,472,700/9,125) in Thailand.

In contrast to economic evaluation using close cohorts, BIA applies open cohorts, which means that the individuals can be included or excluded along a time horizon by incorporating disease incidence and mortality rate in the analysis. Based on the findings of the national cross-sectional study of 36,793 patients with T2D from 1,018 healthcare facilities across Thailand (7), 41.22% undertook at least two oral antidiabetic drugs; 25.52% could not control HbA1c to target 3 times consecutively; and 22.80% received insulin treatment. The estimated incidence of insulin users was 2.4% (22.80% × 41.22% × 25.52%). Therefore, the number of yearly new insulin users was determined by multiplying the number of patients with T2D and the estimated incidence, summing up to 72,487 patients.

#### 2.1.2 Mortality rate of patients with T2D

Risk of death from T2D was reported by the Department of Disease Control, Ministry of Public Health (8) and the mortality rate of the general Thai population was obtained from the Public Health Statistics, Ministry of Public Health (9). Compared with non-insulin users, insulin users indicated a hazard ratio (95% confidence interval [CI]) for the mortality rate of patients with severe hypoglycemia of 3.6 (3.1–4.3) (10). We derived the mortality rate of patients with T2D and severe hypoglycemia by multiplying the hazard ratio and mortality rate of the Thai population at the age of 60 years. This rate was eventually converted to risk. The risk of death from having T2D and the risk of death from having severe hypoglycemia were incorporated in the BIA model to estimate the eligible population.

#### 2.1.3 Prevalence of severe hypoglycemia

The yearly prevalence of severe hypoglycemia was 15% for patients with T2D and insulin treatment (11). Compared with NPH insulin, the risk of severe hypoglycemia for patients with IGlar was reduced by 46% (*p* = 0.0442) (12).

#### 2.1.4 Costs

The cost of IGlar was determined using a pharmaceutical company. IGlar 100 IU/mL of 3 mL cost 0.83 THB per unit while IGlar 100 IU/mL of 10 mL and 300 IU/mL of 1.5 mL cost 1.28 THB per unit. The 0.83 THB per unit was employed in the base-case analysis, and the higher cost was used in sensitivity analysis. The cost of NPH insulin was obtained from the Drug and Medical Supply Information Center, Thailand Ministry of Public Health (13). The costs of NPH insulin 100 IU/mL of 3 mL and 10 mL were 0.26 THB per unit and 0.13 THB per unit, respectively. The 5-year market share of both dosage forms was 46% vs. 54%. Therefore, the average cost of NPH insulin was equal to 0.19 THB per unit (0.26 × 0.46 + 0.13 × 0.54). The total yearly cost of insulin was estimated from the daily-dose multiplied by 365 days. Based on the expert’s opinion, the daily insulin dose in Thailand was found to be 25 units/day/patient.

The cost per event of severe hypoglycemia was obtained from the previous cost-effectiveness of IGlar in Thailand (14). The total cost of severe hypoglycemia was estimated from the cost per event of severe hypoglycemia and the prevalence of severe hypoglycemia. All costs were adjusted to 2022 values using the medical care component of the Thai consumer price index (15). The costs were converted at a rate of 34.54 THB/USD as on December 30, 2022 (16).

### 2.2 Study perspective

This study considered the payer’s perspective; therefore, only the direct medical costs—the costs of NPH insulin and IGlar and the cost associated with severe hypoglycemia—were included. For base-case analysis, the cost of severe hypoglycemia was excluded.

### 2.3 Data analyses

#### 2.3.1 Base-case analysis

The BIA was performed over a period of 5 years based on the Thailand Health Technology Assessment guidelines (17). The details of BIA inputs were shown in Table 1. The total budget was calculated from the following two scenarios: Scenario 1 was that all patients with T2D and severe hypoglycemia received NPH insulin and scenario 2 was the replacement of NPH insulin by IGlar at the rate of 20% in year 1. The uptake rate of IGlar increased by 20% each year until achieving 100% in year 5. The total budget considered only the cost of insulin and the combination of costs of insulin and severe hypoglycemia. The net budget impact (NBI) was the difference in the total budget between scenarios 1 and 2. The results were reported as the yearly cost of NBI and the average cost of NBI.

**Table 1:** Budget impact model inputs.

Variable	Value	Reference
Number of type-2 diabetes cases	3,022,674	Public Health Statistics (9)
Mortality rate of type-2 diabetes	2.5%	Department of Disease Control (8)
Mortality rate of Thai population at the age of 60 years	1.2%	Public Health Statistics (9)
Hazard ratio of mortality rate from severe hypoglycemia (insulin users vs. non-insulin users)	3.6	Akirov A, et al. (10)
Prevalence of severe hypoglycemia	15%	Zammitt NN, et al. (11)
Risk reduction of severe hypoglycemia by insulin glargine compared with NPH insulin	46%	Rosenstock J, et al. (12)
Cost of insulin glargine (THB per unit)	0.83	Industry
Cost of NPH insulin (THB per unit)	0.19	Drug and Medical Supply Information Center (13)
Cost of severe hypoglycemia THB (USD)	29,119 (843.06)	Permsuwan U, et al. (14)

NPH, isophane protamine insulin; THB, Thai baht; USD, US dollar.

#### 2.3.2 Sensitivity analysis

Deterministic sensitivity analysis was carried out to assess the impact of parameter uncertainty. The key parameters, such as the uptake rate of IGlar and the cost of IGlar, varied. The 100% uptake rate of IGlar was applied from the first year. The cost of IGlar increased from 0.83 THB per unit to 1.28 THB per unit with varying IGlar uptake starting at 20 or 100%.

## 3 Results

### 3.1 Base-case results

When IGlar replaced NPH insulin, the total budget would increase depending on the uptake rate of IGlar. In this study, the starting uptake rate of IGlar was 20% and increased by 20% each year. The NBI was approximately 29.1 to 361.1 million THB (0.8–10.5 million USD) from years 1–5, with a yearly average cost of 174.9 million THB (5.1 million USD) when only the cost of insulin was considered. The total budget incurred by the use of NPH insulin only (scenario 1) was higher than that of the uptake of IGlar (scenario 2). This resulted in a negative NBI of 37.3–463.1 million THB or 1.0 to 13.4 million USD with a yearly average cost of 224.3 million THB (6.5 million USD). The results are shown in Table 2.

**Table 2:** Net budget impact from insulin costs with and without the cost of severe hypoglycemia.

Year	Total budget from cost of insulin THB (USD)		NBI THB (USD)	Total budget from costs of insulin and severe hypoglycemia THB (USD)		NBI THB (USD)
	Scenario 1	Scenario 2		Scenario 1	Scenario 2	
	NPH only	NPH + IGlar		NPH only	NPH + IGlar	
1	42,201,022 (1,221,801)	71,258,041 (2,063,059)	29,057,019 (841,257)	763,041,784 (22,091,540)	725,781,452 (21,012,781)	−37,260,331 (−1,078,759)
2	58,923,562 (1,705,951)	140,065,818 (4,055,177)	81,142,256 (2,349,226)	1,065,404,053 (30,845,514)	961,353,899 (27,833,060)	−104,050,154 (−3,012,454)
3	74,927,544 (2,169,298)	229,698,989 (6,650,231)	154,771,445 (4,480,934)	1,354,773,972 (39,223,334)	1,156,307,803 (33,477,354)	−198,466,169 (−5,745,981)
4	90,243,842 (2,612,734)	338,789,199 (9,808,604)	248,545,357 (7,195,870)	1,631,709,816 (47,241,164)	1,312,995,695 (38,013,772)	−318,714,121 (−9,227,392)
5	104,902,007 (3,037,117)	466,047,242 (13,492,972)	361,145,235 (10,455,855)	1,896,745,869 (54,914,472)	1,433,642,927 (41,506,744)	−463,102,941 (−13,407,728)
Average	74,239,595 (2,149,380)	249,171,858 (7,214,009)	174,932,262 (5,064,628)	1,342,335,099 (38,863,205)	1,118,016,355 (32,368,742)	−224,318,743 (−6,494,463)

IGlar, insulin glargine; NBI, net budget impact; NPH, isophane protamine insulin; THB, Thai baht; USD, US dollar.

^1^Net budget impact = Total budget (scenario 2) – Total budget (scenario 1).

### 3.2 Sensitivity analysis results

**Figure 2:**
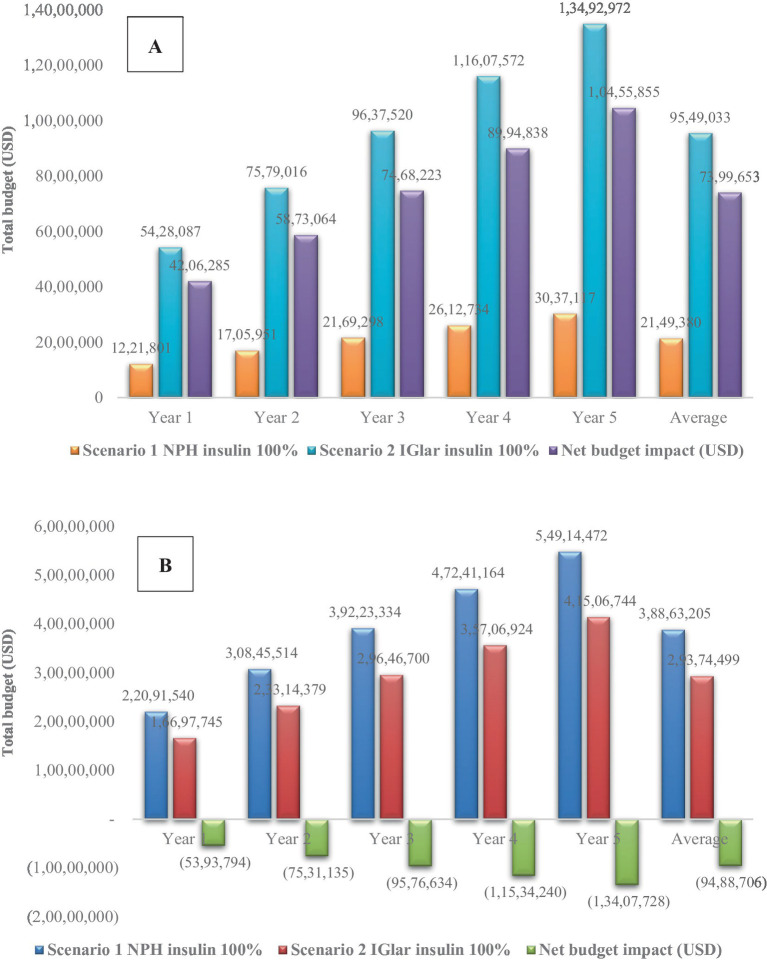
Sensitivity analysis with 100% uptake of insulin glargine considering **(A)** only the cost of insulin and **(B)** both costs of insulin and severe hypoglycemia. IGlar, insulin glargine; NPH, isophane protamine insulin; USD, US dollar.

With an increase in the uptake rate of IGlar to 100% from the first year, the cost of NBI was approximately 4.2–10.5 million USD—that is, with a yearly average cost of 7.4 million USD. When the cost of severe hypoglycemia was included, the NBI would become negative, indicating less total budget of IGlar adoption (scenario 2) compared with no IGlar adoption (scenario 1). The yearly average cost saving was 9.5 million USD or 327.7 million THB. All results are shown in Figure 2.

**Figure 3:**
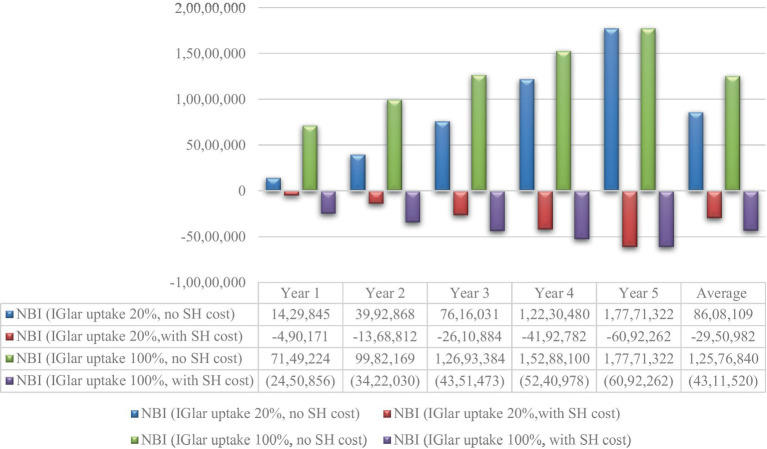
Net budget impact for the cost of glargine 1.28 THB per unit. IGlar, insulin glargine; NPH, isophane protamine insulin; SH, severe hypoglycemia; USD, US dollar.

Although the cost of IGlar increased from 0.83 THB per unit to 1.28 THB per unit, negative NBI was still observed with the inclusion of the cost of severe hypoglycemia. The cost saving was found for the starting IGlar uptake of 20%. However, discarding the cost of severe hypoglycemia, NBI would be substantial depending on the uptake rate of IGlar. The yearly average cost saving of NBI was 8.6 million USD for starting 20% IGlar uptake and 12.6 million USD for 100% IGlar uptake. All results are shown in Figure 3.

## 4 Discussion

The BIA study represents an important tool to support informed decision-making to estimate the financial impact for a specified population of implementing a new health intervention or technology (17). The findings of this study showed that replacing NPH insulin with IGlar would increase the NBI due to a higher unit cost of IGlar compared to that of NPH insulin. The extent of the NBI depends on the rate of IGlar uptake. Specifically, the yearly average cost of NBI was equal to 174.9 million THB (5.1 million USD) when starting with a 20% uptake of IGlar, while it increased to 255.5 million THB (7.4 million USD) with 100% uptake.

However, when we factored in the costs associated with severe hypoglycemia, the narrative shifted dramatically. The introduction of IGlar leads to significant cost savings, with the NBI transitioning to a negative value, indicating that the expenses related to treating severe hypoglycemia effectively offset the higher costs of IGlar. Specifically, the analysis shows a yearly average cost saving of approximately 224.3 million THB (6.5 million USD) when the costs of severe hypoglycemia treatment are included. This underscores the critical importance of considering broader health outcomes in BIA analysis.

In addition, the unit cost of IGlar was a significant factor influencing the analysis. The cost of NBI increased substantially due to the high acquisition cost of IGlar. The yearly cost of NBI increased by approximately 122.4 million THB (3.5 million USD) when the cost of IGlar increased from 0.83 THB per unit to 1.28 THB per unit. Clinical evidence demonstrates that IGlar effectively reduces overall symptomatic and nocturnal hypoglycemia (12). Consequently, the cost of severe hypoglycemia was incorporated into the BIA analysis, resulting in a negative NBI. Reducing the occurrence of severe hypoglycemic events was crucial in offsetting the additional cost associated with IGlar treatment—this benefit persisted even when the unit cost increased to 1.28 THB per unit.

The findings of our study align with other studies that have evaluated the economic impact of IGlar in various healthcare settings. For instance, a survey conducted in the US found that the transition to IGlar was associated with lower overall healthcare costs due to fewer hypoglycemic events and related complications.

This study revealed several strengths; the study was conducted at the request of the Health Economic Working Group (HEWG), working under the subcommittee for the development of the NLEM in Thailand and was conducted after the cost-effectiveness study of IGlar in Thailand (14). Accordingly, some inputs were obtained from the related cost-effectiveness study. This would help the findings of this BIA study to be relevant to the previous cost-effectiveness study and could inform decision-makers when developing reimbursement policies within the resource constraints of the healthcare system. In addition, this BIA study is the first in Thailand to evaluate the financial impact of changing the adoption rate of IGlar use among patients with T2D with severe hypoglycemia. The meeting with an endocrinologist and health economist to validate the BIA model and inputs was established. The suggestions received were considered for the study’s quality improvement. Although no randomized controlled trial has compared IGlar with NPH insulin in Thailand, the risk reduction of severe hypoglycemia by IGlar compared with NPH insulin was based on the results of a meta-analysis of randomized controlled trials, which is classified as the highest level of evidence.

Despite the strengths of this study, including the robust data inputs derived from previous cost-effectiveness analysis, there are several limitations that warrant consideration. First, the assumption that different formulations of IGlar have equivalent clinical efficacy and safety may oversimplify the analysis. The patient-level meta-analysis showed that IGlar 300 IU/mL provided comparable glycemic control to IGlar 100 IU/mL with less severe hypoglycemia at any time of day and less nocturnal hypoglycemia (18). Future studies could benefit from examining the specific impacts of IGlar 300 IU/mL in reducing severe hypoglycemia compared with IGlar 100 IU/mL, potentially enhancing the economic argument for adopting the higher concentration formulation. Second, except for the cost of severe hypoglycemia, this BIA model did not capture the effect of severe hypoglycemia concerning other aspects such as other hypoglycemia-related complications. Third, this study did not include indirect costs and patients’ health-related quality of life owing to the perspective of the study. Finally, in the absence of actual data on the adoption rate of IGlar, we assumed an initial adoption rate of 20% based on expert opinion.

## 5 Conclusion

The yearly NBI of IGlar adoption in the treatment of patients with T2D and severe hypoglycemia from NPH insulin was 174.9 million THB (5.1 million USD). A lower rate of severe hypoglycemia with IGlar than those treated with NPH insulin generates cost savings, resulting in significantly reduced additional costs of IGlar. Therefore, the yearly NBI became negative.

## Data Availability

The raw data supporting the conclusions of this article will be made available by the authors upon reasonable request, without undue reservation.

## Author disclaimer

This paper represents the views of the authors. This study was conducted at the request of the HEWG. This manuscript is one part of the project “Budget impact analysis of insulin glargine sterile solution for patients with type 2 diabetes having severe hypoglycemia from conventional insulin treatment” which was used to support the policy-making process under the Subcommittee for the Development of the NLEM in Thailand through the HEWG but the HEWG is not responsible for the study findings or their dissemination.
